# Human Ascaris infection is associated with higher frequencies of IL-10 producing B cells

**DOI:** 10.1371/journal.pntd.0012520

**Published:** 2024-09-23

**Authors:** Josefina Zakzuk, Juan F. Lopez, Cezmi Akdis, Luis Caraballo, Mübeccel Akdis, Willem van de Veen

**Affiliations:** 1 Institute for Immunological Research, University of Cartagena, Cartagena, Colombia; 2 Swiss Institute of Allergy and Asthma Research (SIAF), University of Zurich, Davos, Switzerland; TOBB Economics and Technology University Faculty of Medicine: TOBB Ekonomi ve Teknoloji Universitesi Tip Fakultesi, TÜRKIYE

## Abstract

**Introduction:**

*Ascaris lumbricoides* has dual effects on the immune system of infected hosts. The IgE response to this parasite has been thoroughly studied, but little is known about cellular responses induced by infection. This study aims to explore the interplay between *A*. *lumbricoides* infection and B cell responses, especially B regulatory cells.

**Methods:**

Participants from Santa Catalina, Bolívar, Colombia, a helminth-endemic town, were screened for soil-transmitted helminthiasis (STH) using stool examinations. Eighteen *A*. *lumbricoides*-infected and 11 non-infected subjects were selected. Blood samples were analyzed for Breg cells and related cytokines, and immunoglobulins specific to the *A*. *lumbricoides* excretory/secretory product, ABA-1.

**Results:**

Infected subjects exhibited higher frequencies of Breg cells, especially those with a higher *A*. *lumbricoides* egg burden. Higher frequencies of different Breg subsets were observed in infected individuals, with CD25^+^CD71^+^CD73^-^ B cells being notably increased in strongly infected individuals. Additionally, *A*. *lumbricoides* infection was associated with reduced levels of circulating ABA-1-specific IgG1 and IgE. IL-10^+^ B cell frequencies correlated inversely with ABA-1-specific IgE.

**Conclusions:**

*A*. *lumbricoides* infection has a significant impact on the immune response, particularly on Breg cell populations and antibody responses. Our findings suggest that *A*. *lumbricoides* infection mediates a dose-dependent immunosuppressive response characterized by an increase in Breg cells and concomitant suppression of ABA-1-specific humoral responses.

## Author summary

*Ascaris lumbricoides*, a common parasitic worm, may modulate the immune system in different ways. While much is known about the IgE antibody response to this parasite, the cellular immune responses remain less understood. Our research question in this study was how *A*. *lumbricoides* infection influences B cell responses, with a focus on regulatory B cells (Bregs).

We conducted our research in a rural town in the North Coast of Colombia, an area where soil-transmitted helminth infections are prevalent. We compared blood samples from 18 individuals infected with *A*. *lumbricoides* and 11 non-infected individuals to analyze Breg cells, related cytokines, and specific antibodies against the parasite’s excretory/secretory product, ABA-1. We observed that individuals infected with *A*. *lumbricoides* have higher frequencies of Breg cells, especially those with a more intense infection (higher counts of parasite eggs in feces). In particular, certain subsets of Breg cells were significantly elevated in those with higher parasite loads. Interestingly, infection was also linked to reduced levels of antibodies specific to ABA-1. Notably, the frequency of IL-10^+^ Breg cells was inversely related to the levels of ABA-1-specific IgE.

In conclusion, *A*. *lumbricoides* infection may induce immunosuppressive responses, marked by an increase in regulatory B cells and a decrease in specific antibody responses. These findings highlight the intricate ways in which this parasite modulates the immune system, which could have implications for understanding immune regulation in parasitic infections.

## Introduction

*Ascaris lumbricoides* is the primary cause of soil-transmitted helminthiasis (STH) in humans. Epidemiological studies have revealed a dual impact of this parasitic infection on allergy development [1–4]. Mild infection is associated with higher risk of asthma and atopy, probably due to an inductive effect on type 2 responses. In contrast, severe ascariasis appears to diminish the risk of asthma symptoms among populations in endemic areas, which could be associated with susceptibility to immunosuppressive effects of helminth secretory products [5]. It is worth noting that the presence of *A*. *lumbricoides*-specific IgE antibodies is associated with an increased risk of asthma. Moreover, the IgE response to ABA-1 [6]—the predominant excretory/secretory (E/S) product from Ascaris spp.—is linked, both, to resistance against the infection [7] and to asthma symptoms in parasitized tropical communities [8].

Helminth infections are considered natural models of immune tolerance, primarily mediated by the induction of regulatory T cells and the production of anti-inflammatory cytokines such as IL-10 and TGF-β [9]. While immunosuppression benefits the parasite’s survival, it might also weaken the immune response to other harmful pathogens, vaccinations, and contribute to immune disorders, including allergies and autoimmunity. However, much of our knowledge on helminth infections comes from murine models, and for certain parasites such as *A*. *lumbricoides*, this remains insufficient to fully understand the human immune response to the infection. Data regarding humans is limited to certain parasites, with a majority of the literature centered on Schistosoma infections [10,11] and various filariae [12]. The best-documented immunosuppressive effects of helminth infections are diminished antigen presentation [13], reduced effector functions of mast cells and basophils [14], and the triggering of regulatory T-cell responses [15].

B cells play a significant role in immunomodulation, mainly associated with the induction of type 2 response-related immunoglobulin isotypes, like IgE and IgG4, and production of anti-inflammatory cytokines such as IL-10 and TGF-β [16,17]. Beyond their role in antibody production, B cells exhibit other functional attributes. Notably, certain B cell subsets, termed Breg cells, can produce IL-10. This has implications in diverse health conditions, including autoimmunity [18,19], allergy [20], cancer [21], and even immune tolerance to bee venom following immunotherapy or repeated natural exposure [22]. The challenge lies in the variety of cellular markers reported for Bregs (IL-10^+^ CD1d^hi^, IL-10^+^CD5^+^, CD5^+^CD1d^hi^ and IL-10^+^CD24^hi^CD38^hi^ cells) [16], complicating the task of consistently identifying specific phenotypes or sub-populations [10,22,23]. B regulatory 1 (BR1) cells, recognized initially through bottom-up transcriptomics, are distinguished by their CD25^hi^CD71^hi^CD73^low^ expression [22]. This marker combination strongly aligns with their functionality, encompassing not just IL-10 production but also the induction and release of specific IgG4 antibodies. In light of these findings, our study aims to evaluate the interplay between antibody responses and Breg cells in the context of an *A*. *lumbricoides* infection in an endemic area of the rural Northern Coast of Colombia, Latin America.

## Results

### *A*. *lumbricoides* infection is associated with greater frequencies of Breg cells

**Fig 1:**
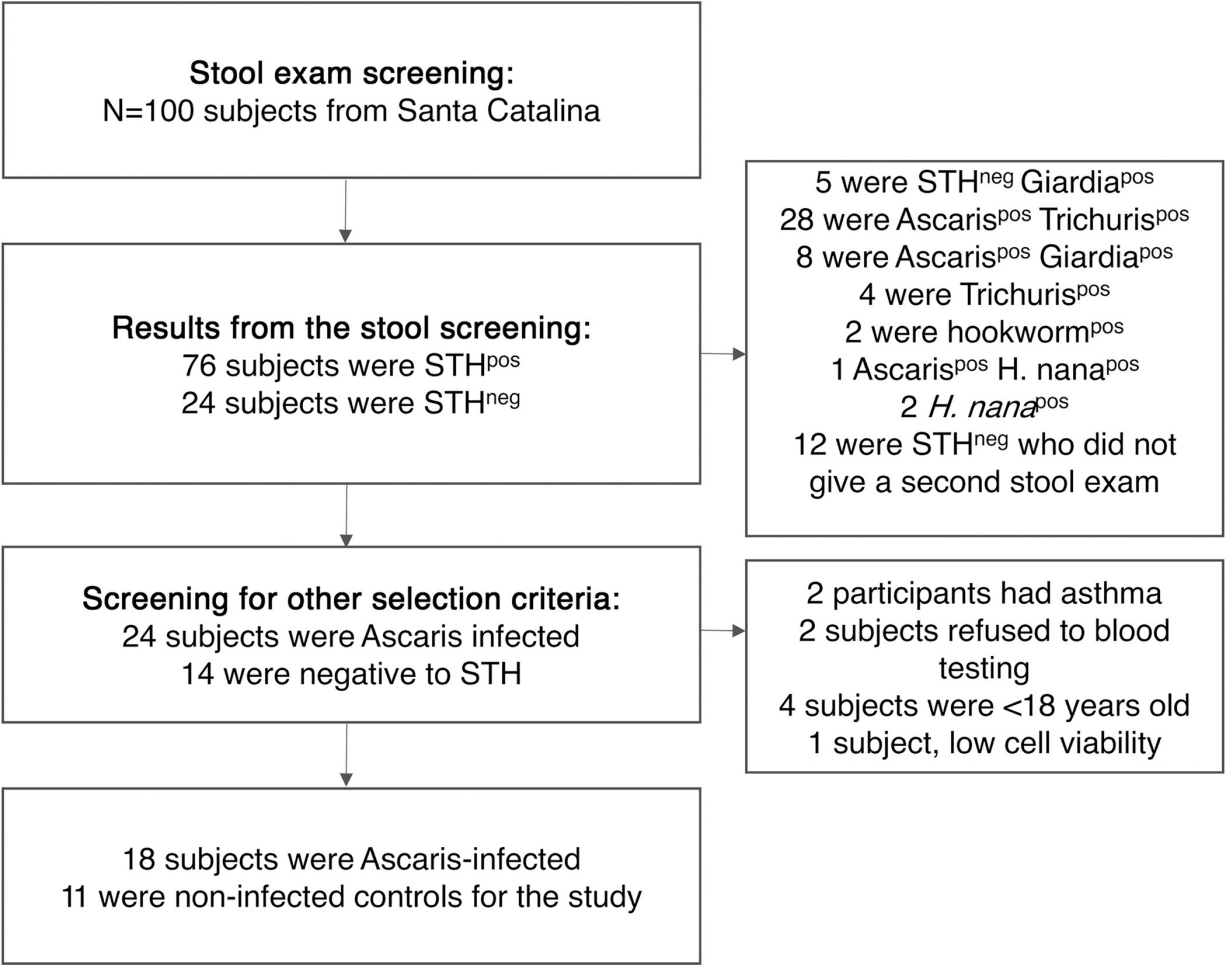
Flowchart of the selection of participants. Abbreviatures: STH^neg^: subjects tested negative for helminth infections. STH^pos^: subjects who tested positive for any helminth. Neg: negative. Pos: positive.

A total of 100 inhabitants of Santa Catalina were screened by stool examination to identify the sample study. A flow chart is presented in Fig 1 with the details of the screening process. Descriptive features of the study groups for cellular analysis are shown in Table 1.

**Table 1:** Descriptive of the sample study.

	Non-infected (n = 11)	Infected subjects (n = 18)
Age [mean ± SD]	36.2 ± 16.1	30.5 ± 16.1
Sex^¶^ [n (%)]	9 (81.8)	11 (61.1)
Egg burden [median (IQR)]	0	Light (<3000): 1800 (1116–2760) Strong (≥3000): 8184 (5124–19680)

^*¶*^ *Females*. *IQR*: *interquartile range*. *Mann Whitney test was used for two-group comparisons*. *Significant differences are highlighted in bold*. *Light and strong categories are based on the median values of egg burden in this population*.

**Fig 2:**
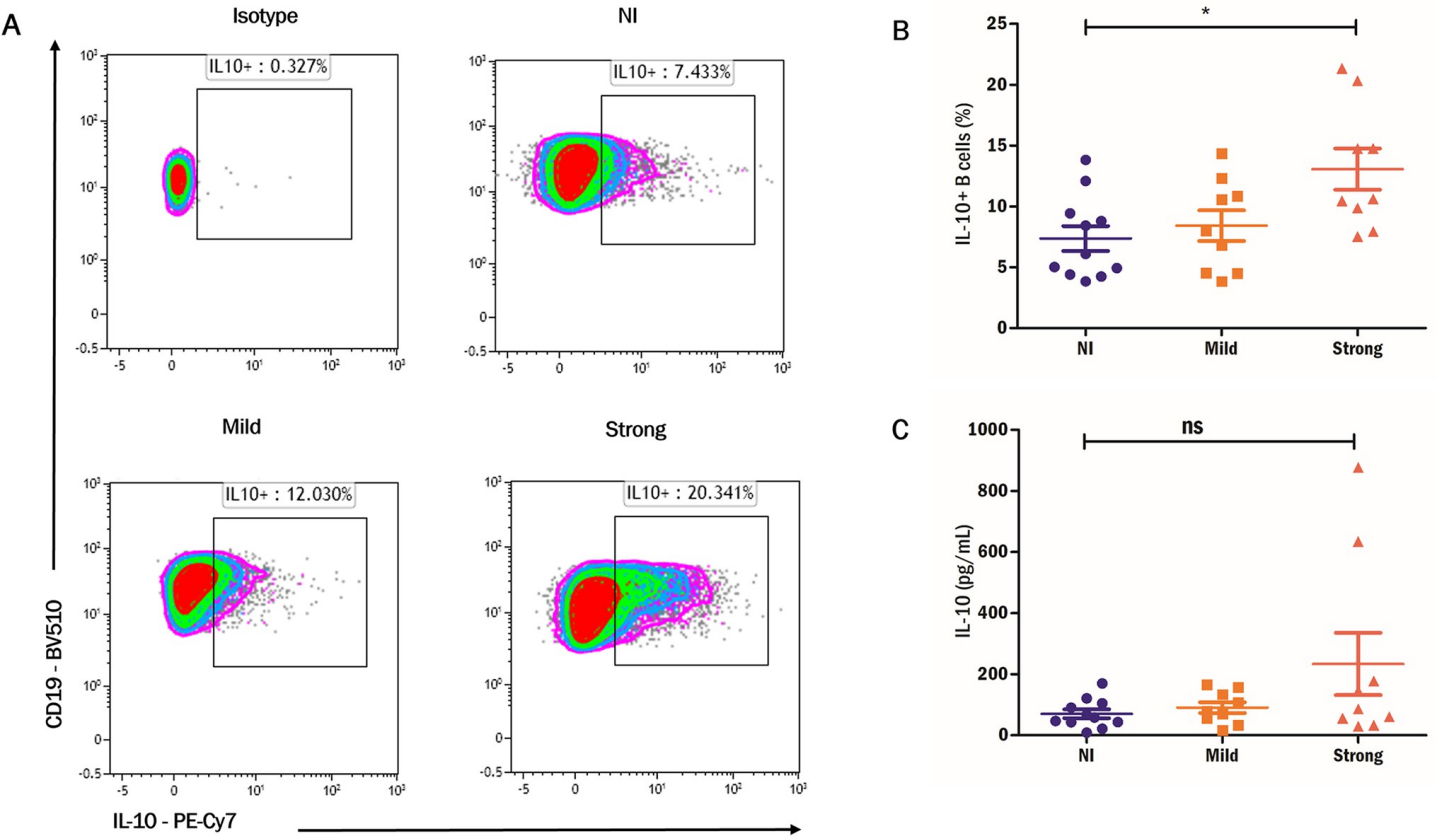
Percentage of IL-10+ B cells are increased in subjects infected with ascariasis. A) Representative density plots of the study groups and isotype control staining. The observed B cell population is gated on live CD3^-^CD19^+^ events. B) %IL-10^+^ B cells are compared among non-infected subjects (NI) and patients with mild or strong egg burden. C) IL-10 levels in the supernatants of 3-day CpG-stimulated PBMC cultures. Kruskal Wallis Test was used for the three group comparisons. Ns: non-significant, *p<0.05.

In regard to Breg cell analysis (**Fig 2A and 2B**), higher relative numbers of IL-10-producing B cells were found in *A*. *lumbricoides* infected subjects than in the NI group, mainly in those with stronger egg burden infection (p = 0.035). There was no difference between sex (men: 9.75 SD ±5.1 vs women 9.33 SD 4.60, p = 1.0). Age was not correlated with IL-10^+^ B cells (rho: -0.18, p = 0.345). Higher levels of IL-10 were also observed in supernatants from CpG-stimulated PBMCs from subjects with stronger egg burden; however, this difference did not achieve statistical significance (**Fig 2C**). Basal production of IL-10 in unstimulated cultures was measured by ELISA, with no differences in supernatant levels among infection groups. To evaluate the capacity of *A*. *lumbricoides* antigens to induce Breg cells, PBMCs were stimulated with ABA-1 and the *A*. *lumbricoides* somatic extract (ASE) for 3 or 6 days. We observed a modest effect in response to stimulation with 1 μM ABA-1, but not the complete extract, on inducing IL-10^+^ B cells at day 6, but not at day 3. Breg cell induction was mainly observed in infected subjects, but not NI controls (**S1 Fig**).

### Different Breg cell subsets are increased in *A*. *lumbricoides* infected patients

**Fig 3:**
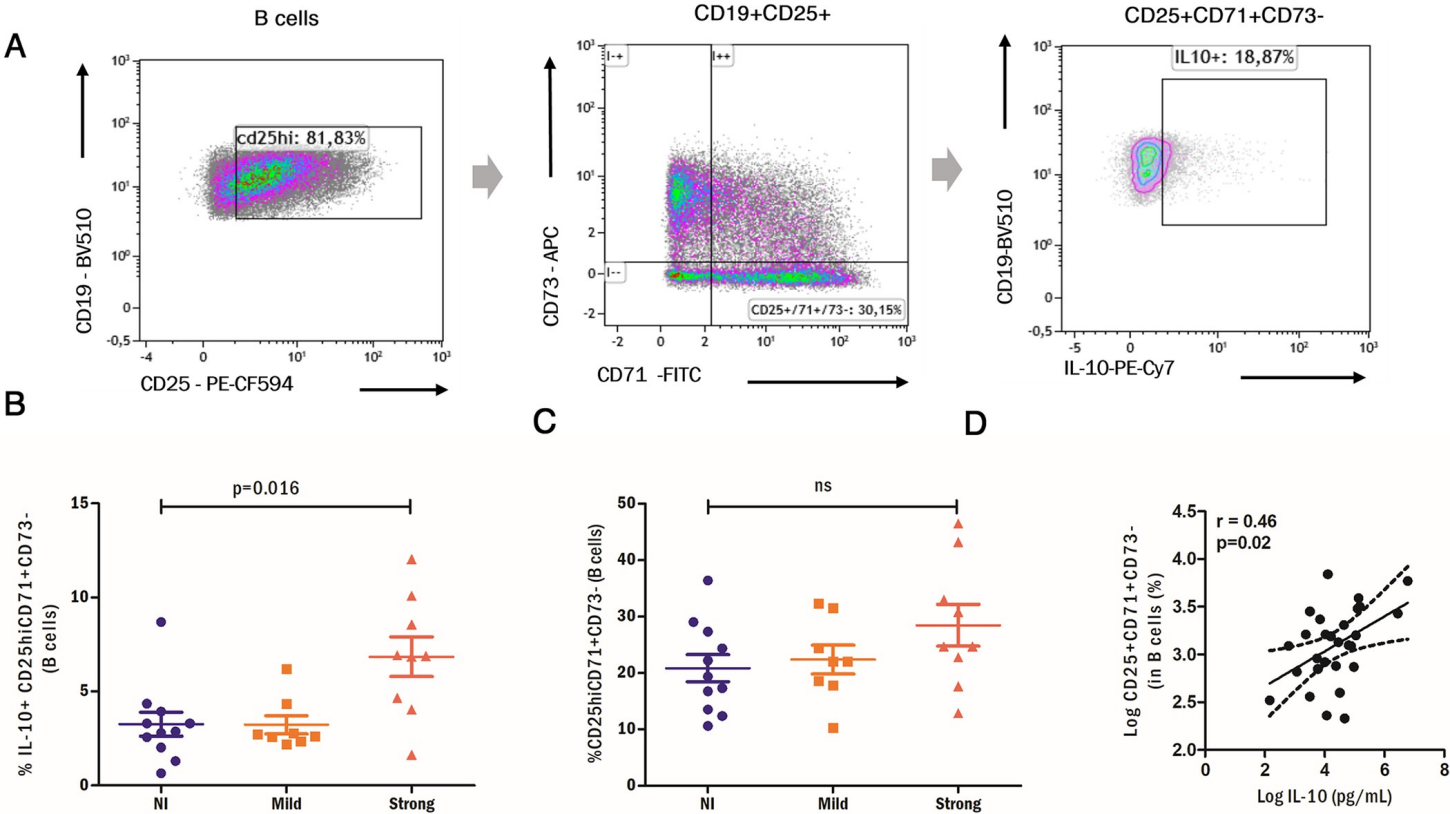
Analysis of IL-10^+^ CD25^+^CD71^+^CD73^-^ B cell frequencies in relation with ascariasis. A. Gating strategy for identification IL-10^+^CD25^+^CD71^+^CD73^-^B cells. The observed starting cell population is gated on live CD3-CD19+ events. B) % CD25^+^CD71^+^CD73^-^ B cells are compared among non-infected subjects (NI) and patients with lower (mild) or higher (strong) *A*. *lumbricoides* egg burden. C) Log-transformed values of CD25^+^CD71^+^CD73^-^ B cells are correlated with IL-10 supernatant levels in CpG stimulated cultures (Spearman test was used, rho value is reported). D) IL-10^+^ CD25^+^CD71^+^CD73^-^ B cell relative numbers are compared between the three groups. Kruskal Wallis test was used for the three group comparisons. *p<0.05, Ns: non-significant.

We aimed to evaluate the relationship of *A*. *lumbricoides* infection with several Bregs subsets previously reported in relation with other helminth infections [10,24]: IL-10^+^ CD1d^hi^, IL-10^+^CD5^+^, CD5^+^CD1d^hi^ and IL-10^+^CD24^hi^CD38^hi^ B cells as well as the CD25^+^CD71^+^CD73^-^ B cell phenotype, first reported by our group to be associated with IL-10 production and inhibition of T cell proliferation [23]. Comparison of NI vs all infected subjects did not yield significant differences in the frequencies of IL-10^+^ CD25^+^CD71^+^CD73^-^ B cells (3.2% in NI vs 5.1% in infected, p = 0.16); however, as shown in **Fig 3B**, when categorized infection intensity, relative numbers of this Breg cell subset were significantly higher in those with stronger egg burden (lower: 3.2%, higher: 6.8%, p = 0.035). The CD25^+^CD71^+^CD73^-^ relative numbers among B cells did not significantly vary among infection groups **(Fig 3C**); however, IL-10 levels in supernatants from CpG stimulated cultures significantly correlated with them but not with other Bregs subsets (**Fig 3D**). We also analyzed whether the frequency of IL-10^+^ B cells could be enriched within the analyzed Breg cell subsets (**S2 Fig**), observing that only the CD25^+^CD71^+^CD73^-^ combination could significantly raise the mean frequency of IL-10^+^ B cell numbers (17.8%) compared to the relative number among total B cells (9.5%).

**Fig 4:**
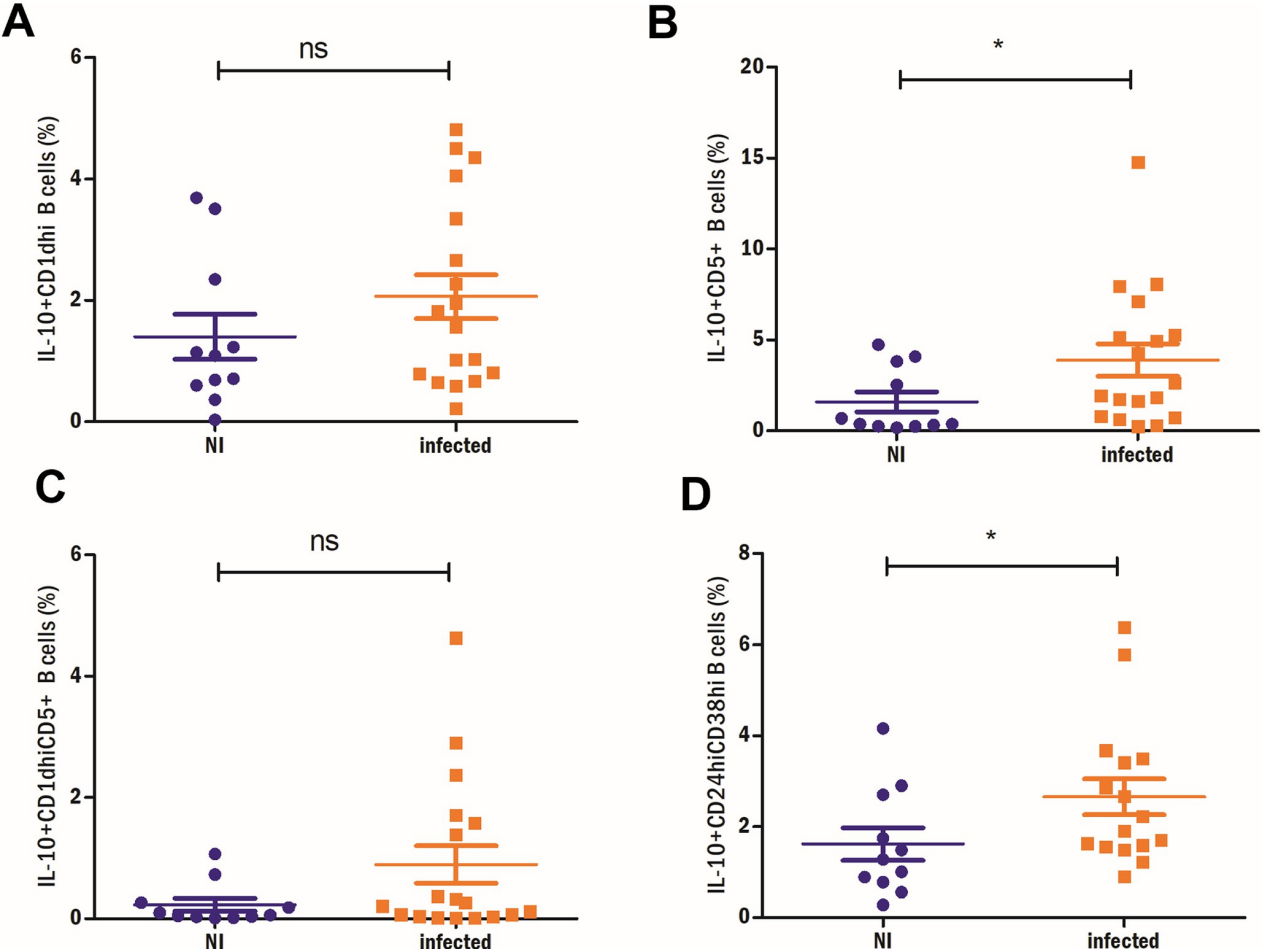
Analysis of other Breg cell subsets in relation with ascariasis. A) IL-10^+^ CD1d^hi^, B) IL-10^+^CD5^+^, C) IL-10^+^ CD5^+^CD1d^hi^ and D) IL-10^+^CD24^hi^CD38^hi^ B cells are compared in non-infected and *A*. *lumbricoides* infected subjects. Mann-Whitney test was used for two-group comparisons. *p<0.05, ns: non-significant.

IL-10^+^ CD5^+^ (1.6% vs 3.8%, p = 0.03) and IL-10^+^ CD24^hi^CD38^hi^ B cells (1.6% vs 2.9%, p = 0.049) were also significantly higher in infected patients (**Fig 4A and 4B**). No differences were found for IL-10^+^ CD5^+^CD1d^hi^ (0.23% vs 0.9%, p = 0.29) and IL-10^+^ CD1d^hi^ B cells (1.4% vs 2.0%, p = 0.17) (**Fig 4**).

### *A*. *lumbricoides* infection is associated with lower ABA-1-specific IgG1 and IgE levels

**Fig 5:**
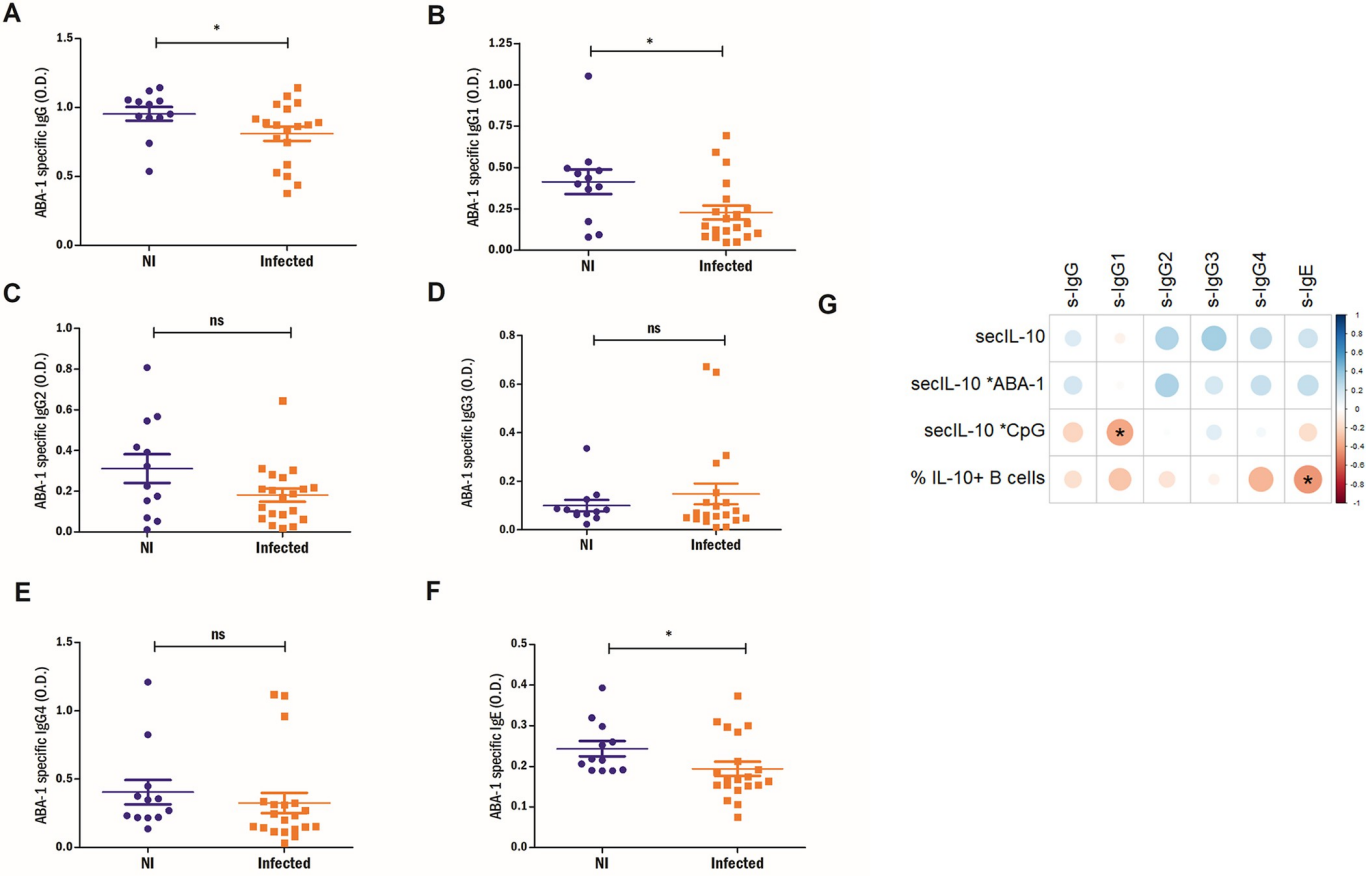
Specific ABA-1 antibody responses in ascariasis. ABA-1 specific A) IgG, B) IgG1, C) IgG2, D) IgG3, E) IgG4 and F) IgE is compared between non-infected (NI) and infected subjects. Mann-Whitney test was used for two-group comparisons. *p<0.05, ns: non-significant. G) Correlogram depicting values for all analyzed isotypes, % IL-10^+^ B cells and IL-10 secreted (secIL-10) levels from supernatants from PBMC cultures stimulated with ABA-1, CpG or only medium. Only significant correlations are highlighted with an asterisk. The scale indicates the Spearman coefficient (rho) from –1 to 1. In the heatmap scale, red and blue colors indicate direct and inverse correlations, respectively.

Median IgG anti-ABA-1 was significantly higher in individuals from Santa Catalina than in European subjects with no history of past *A*. *lumbricoides* infection (**S3 Fig**). ABA-1-specific antibodies of different isotypes (IgG, IgG1 and IgE) were significantly lower in *A*. *lumbricoides* infected subjects (**Fig 5A, 5B and 5F**). No differences were observed for anti-ABA-1 specific -IgG2, -IgG3 or -IgG4 (Fig 4C–4E). Notably, ABA-1 specific IgE inversely correlated with IL-10+ producing B cell numbers (r = -0.42, p = 0.02). Specific anti-ABA-1 of other isotypes did not show correlation with IL-10 production by B cells (**Fig 5G**). Anti-ABA-1 specific IgG1 correlated with IL-10 production measured in supernatants from CpG stimulated cultures (r = 0.38, p = 0.045).

### *A*. *lumbricoides* infection is associated with increased production of type 2 cytokines

T cell cytokine responses were also analyzed in PBMC cultures after polyclonal T cell stimulation (anti-CD2, anti-CD3, and anti-CD28) for 3 days. Higher levels of IL-4 (p = 0.035), IL-5 (p = 0.06) and IL-9 (p = 0.007) were found in supernatants from infected subjects versus NI participants. No differences in regard to IL-10 were detected (See all comparisons in **S4 Fig**).

## Discussion

Studies on human Breg cells associated with helminth infections are limited [24,25]. For the first time, we report that an increased number of Breg cells is linked to *A*. *lumbricoides* infection, the leading cause of STH globally [26]. Most research about the immune effects of ascariasis has been related to the antibody response, blood cytokine responses, but little is known about B cell immunobiology [2,5]. Our study contributes to the knowledge of additional anti-inflammatory mechanisms associated with *A*. *lumbricoides* infection. Particularly, this infection is a well-known inducer of type 2 responses and also, under certain conditions, a risk factor for asthma and atopy [5,27,28]. However, there is also evidence of its potential to reduce inflammatory responses and protect from allergy in the context of severe infections [1]. Identification of cellular or humoral mechanisms behind these effects have not been completely elucidated. Here, we report that, although *A*. *lumbricoides* infection was associated with type 2 T cell responses manifested by significantly higher IL-4 and IL-9 levels, stronger infection was mostly associated with higher numbers of IL-10 producing B cells as well as of several Breg cell subsets previously linked to helminthiasis.

In *A*. *lumbricoides* infected subjects, we observed increased frequencies of IL-10^+^ B cells upon stimulation with the TLR-9 ligand CpG2006, a known inducer of this cytokine [22], when compared to NI subjects. This raises the question whether there is a direct causal relationship between *A*. *lumbricoides* infection and the development of Breg cells. Interestingly, the fact that B cells produced IL-10 post-stimulation with ABA-1 suggests that a helminth secretory product might be responsible for this effect during the infection. However, the higher frequencies of B cells producing IL-10 in infected subjects implies that these individuals could be more prone to the immunosuppressive effects of the parasite’s components, suggesting individual predisposition to immunomodulation.

Active infection seems to be necessary for the observed effects on B regs, since most people had been previously infected with *A*. *lumbricoides*, as suggested by the high seroprevalence of anti-ABA-1 response. Interestingly, ABA-1 is a fatty acid binding protein (FABP). This biochemical function has also been observed in other helminth molecules known to induce IL-10 production. For instance, Chayé et al. discovered that immunization with Sm14, an FABP from *Schistoma mansoni*, induced Bregs development in mice [29].

Parasite derived molecules may employ different mechanisms to promote IL-10 production by B cells. Induction of regulatory T cell response may indirectly augment regulatory B cell functions, but it is also possible that they act directly on B cell receptors activating signaling pathways conducing to IL-10 production. Ascariasis was associated with higher frequencies of CD5^+^ B cells. The function of this molecule is best characterized in mice, but molecular studies in humans linked its activation with IL-10 expression promoting pathways [30]. Also, non-protein molecules, such as phosphorylcholine residues and sphingolipids [31] may induce IL-10 production and other immunosuppressive effects such as inhibition of T cell proliferation, modulation of cytokine production and induction of Breg cells [32] and their importance in ascariasis immunobiology must be explored.

IL-10^+^CD5^+^ B cells were more frequent in *A*. *lumbricoides* infected subjects than in NI subjects. Murine CD5+ B cells or B1 cells [30] have functions related with B cell survival prior to the antigenic encounter, IL-10 secretion and producing natural/polyreactive antibodies. In humans, its role is less understood; B1 cells are not defined by CD5 expression; however, B cells expressing this marker are more abundant in natural states of tolerance or helminth infection. Reduced CD19^+^CD5^+^ Bregs have been associated with milk allergy in children [33]. In addition, a newly identified population of human cord blood CD5^hi^ cells was found to secrete IL-10 upon infection by the respiratory syncytial virus and subsequently, more adverse clinical outcomes [34]. In contrast to other reports about the role of Breg cells in different helminthiasis, *A*. *lumbricoides* infection was not associated with higher numbers of CD1d^hi^ B cells or IL-10^+^CD1d^hi^ B cells [10,11,35]. Conversely, CD1d^hi^CD5^+^ cells were more frequent in *A*. *lumbricoides* infected subjects, as it has also been reported for other helminth infections [10,36].

A novel finding of this study was the association of helminth infection with the frequency of IL-10^+^CD25^+^CD71^+^CD73^-^ B cells, a phenotype that had been previously linked to bee venom tolerance in beekeepers [22] and allergen-specific immunotherapy for house dust mite [37]. Also, this marker combination led to the highest enrichment of IL-10^+^ B cells among cases with strong *A*. *lumbricoides* infection. This Breg phenotype was discovered by our research group using a bottom-up transcriptomics approach that revealed that this set of markers could efficiently enrich IL-10 producing B cells [22]. As previously demonstrated, CD25^+^CD71^+^CD73^-^ B cells inhibited T cell proliferation *in vitro* and selectively produced IgG4 [22], an isotype associated with tolerance and anti-inflammatory states in scenarios such as food tolerance [38,39] and higher burden of schistosomiasis [40]. However, we did not observe a relationship between IgG4 and *A*. *lumbricoides* infection status. As also reported by McSharry *et al*, we found higher levels of specific IgE to ABA-1 in uninfected individuals, in contrast to ABA-1 specific IgG4 [7].

ABA-1-specific IgG1 and IgE levels were reduced in infected subjects compared to NI controls, whereas IgG2 and IgG4 levels did not significantly differ between the groups. It should be noted that the NI group most likely had prior infections with *A*. *lumbricoides* given the fact that they were living in an *A*. *lumbricoides* endemic area, and their high levels of ABA-1- specific IgG antibodies compared to European non-infected controls. The lower ABA-1-specific IgG1 and IgE levels in the infected group might reflect active suppression of ABA-1specific humoral responses during acute infection. This suppression appears to be less pronounced for IgG2 and IgG4. This might be due to the tolerogenic responses that are induced by the parasite, which are known to promote IgG4 production [41]. Moreover, a recent study demonstrated that in besides IgG4, IgG2 responses were also associated with tolerance induction in response to allergen immunotherapy [42,43].

*A*. *lumbricoides* infection is still a prevalent problem in rural areas of developing countries [44], especially in South America. Although mass or targeted deworming strategies may reduce the prevalence of STH [45], predisposed individuals tend to reinfect if ecological niche are not modified through sanitary interventions. In the rural areas of the North Coast of Colombia, poverty conditions still arise and access to tap water and sewage implementation is limited; then, STH maintain as chronic public health problems [5]. Understanding the effects of infection on immune responses may have public health relevance for different reasons. One, in endemic areas, an immunosuppressive state derived from chronic and intense ascariasis may have detrimental effects on vaccination or defense against microbial infections [46]. On the other hand, understanding how parasite modulates the immune system may help identifying mechanisms that could be of relevance to treat inflammatory chronic diseases [47,48]. Also, collateral effects of reducing STH may be the loss of natural mechanisms of immune regulation [49]; then, there is a need of more integrated programs that take care of social determinants and chronic inflammatory disease risk in communities or countries that face transitions from high to low burden of helminthiasis [50,51].

Our findings provide valuable insights into the modulation of the immune system by *A*. *lumbricoides*, particularly regarding regulatory B cells (Breg) and specific humoral responses. In particular, we observed that *A*. *lumbricoides* infection is associated with increased frequencies of different Breg subsets, suggesting a potential immune evasion strategy by the parasite [52]. This increase in Breg cells could play a key role in suppressing parasite-specific immune responses, as evidenced by the reduced levels of ABA-1-specific IgG1 and IgE. Also, the identification of an inverse correlation between IL-10^+^ B cell frequencies and ABA-1-specific IgE levels highlights a potential mechanism of immune regulation that the parasite may exploit to ensure its survival and persistence in the host. These data not only expand our knowledge of helminth-induced immunomodulation but also have significant implications for the development of new therapeutic and vaccination strategies against helminth infections.

Several limitations must be recognized in this study. Sample size is small, and this decrease the power to detect associations and limit the assessment of potential confounders in multivariate models. Additionally, stool examinations have sensitivity limitations in detecting STH [53,54]. To address this, we performed two consecutive stool exams to increase sensitivity [54]. It is important to highlight that despite the possibility of false negatives due to infected cases with low egg burdens, significant differences were still detected among groups. This may also be interpreted as a strong effect on B cells despite these limitations. Since polyparasitism is frequent in endemic areas, in spite we exclude cases with co-infection with another helminth, we cannot ascertain that other helminthiasis, not detected by fecal exam, can influence results [55]. For example, toxocariasis is considered a neglected disease in Colombia with an incidence rate of 12 per 100,000 inhabitants of Bolívar, the Department were Santa Catalina is located [56]. Also, although different studies have demonstrated that analyzed Breg populations are functionally active and inhibit T cell responses, we did not evaluate their impact on antigen-specific T cell responses related with *A*. *lumbricoides*. Additionally, our study was limited to IL-10 producing B cells, as a readout for quantifying Breg cells, while we have not investigated the induction of other Breg cell cytokines or effector molecules, like IL-35 [57], TGF-β or Granzyme B [58], or specific surface molecules which may have a regulatory function, such as PD-L1 or FasL [59].

In conclusion, different subsets of IL-10 producing B cells are more frequent in subjects with *A*. *lumbricoides* infection, particularly those with a greater helminth egg burden. This IL-10 producing activity in B cells is inversely correlated with specific IgE production towards ABA-1, a trait that has been previously linked with resistance to infection. Through this study, we provide evidence that infection by *A*. *lumbricoides* can induce anti-inflammatory mechanisms, supporting this dual effect observed in highly exposed populations. Additionally, it is highlighted that the population of IL-10^+^CD25^+^CD71^+^CD73^-^ B cells represent a phenotype of regulatory B cells present in various contexts of immunomodulation induced by environmental stimuli.

## Material and methods

### Ethics statement

The study was approved by the University of Cartagena Ethics Committee (#17-05-2012). Formal written consent was obtained from all participants.

### Subjects

For this study, subjects living in Santa Catalina, Bolívar, a helminth-endemic rural town [5] located in the northern coast of Colombia were invited to participate in a screening of STH infection by stool examination. Adult participants with no antecedents of allergic or autoimmune diseases were selected. Helminth or/and *Giardia lamblia* co-infection and receiving anti-helminthic in the last 3 months were exclusion criteria. Thirty mL of blood was drawn from 18 *A*. *lumbricoides*–infected patients and 11 non-infected subjects. Non-infected (NI) controls must have two negative stool examinations to *A*. *lumbricoides* and any other parasite. Of note, as we had previously performed a prevalence STH study in the town [5], searching for new participants was partially guided by previous information on STH status. Thus, frequency of STH in this sample study is not representative of the actual distribution of stool parasites in Santa Catalina.

Infection status was determined by stool parasitological analyses. Helminth egg burden was determined by Kato Katz method and expressed as egg per gram of feces (e.p.g.) in two stool samples obtained by spontaneous evacuation on two different days within one-week period. Infected subjects were further sub-categorized into mild or lower (<3000 e.p.g.) or strong (≥3000 e.p.g.) egg burden. This cut-off was arbitrarily set on 3.000 e.p.g., as the median value for egg counts.

### Isolation of Peripheral Blood Mononuclear Cells (PBMCs)

Blood samples were collected in heparinized tubes (BD Vacutainer REF 367526, Franklin Lakes, NJ.). Subsequently, the samples were diluted in PBS and Ficoll Histopaque (Sigma Aldrich, Ref 10771) in a 1: 1: 1 ratio and centrifuged to visualize and extract the mononuclear cell layer. Cells were washed three times with 2mM PBS/EDTA and resuspended in 1 mL of supplemented RPMI 1640 (Sigma Aldrich, Ref 8758) and 1 mL of freezing medium (80% inactivated FBS and 20% DMSO). The samples were stored in cryovials, refrigerated, and stored at −80°C until analysis.

### Cell cultures

Two million PBMCs were cultured in 24-well plates at a density of 1x10^6^/mL and stimulated with 1 μM of TLR-9 ligand (ODN 2006, 5’-tcgtcgttttgtcgttttgtcgtt-3’) and incubated for 72 hours in 5% CO2 at 37°C (22). Cells were restimulated 25 ng/ml phorbol 12-myristate 13-acetate (PMA, P-8139; Sigma-Aldrich, Buchs, Switzerland) and 1 μg/ml Ionomycin (I-0634; Sigma-Aldrich, Buchs, Switzerland) in the last four hours of culture. Brefeldin A (B-7651; Sigma-Aldrich, Buchs, Switzerland) at 1 μg/mL was used for intracellular detection of cytokine production. Supernatants were collected for cytokines measurement. Cells were also stimulated with other conditions, such as ABA-1 at 1 μM and *A*. *lumbricoides* to evaluate its potential to induce IL-10 production in B cells. To evaluate T cell-related cytokines, PBMCs were stimulated with anti-CD3/CD2/CD28 for three days.

### Flow cytometry

After stimulation, cells were washed in PBS and stained with fixable viability dye eFluor 780 for 30 min at 4°C (65-0865-14; eBioscience, Vienna, Austria) followed by staining with a battery of fluorescent coupled antibodies targeting B reg cell markers: CD71, CD1d, CD25, CD38, IL-10, CD73, CD5, CD24, CD19 and exclusion lineage markers CD3, CD14 and CD16. Before intracellular staining for IL-10 detection, cells were fixed and permeabilized with fixation/permeabilization solution for 20 min at 4°C (BD Cytofix/ Cytoperm Fixation/Permeabilization solution kit, 554714, Allschwil, Switzerland). Samples were acquired with a Gallios Flow cytometer (Beckman Coulter, Brea, CA, USA) with a standardized gating strategies and analyzed using Kaluza software (Beckman Coulter, Brea, CA, USA). Monoclonal antibodies used for cell phenotyping are shown in **S1 Table**.

### Multiplex Cytokine determination

Cytokine levels (IFN-gamma, IL-4, IL-5, IL-9, IL-13, IL-17A, IL-10, IL-22, IL-21 and TNFα) were measured in PBMC by using a Multiplex Cytokine Immunoassay with Magnetic Beads (Milliplex, Schaffhausen, Switzerland) in a Bio-Plex 200 (Bio-Rad, Hercules, US).

### Measurement of antibody responses

ABA-1 was selected as the representative antigen of *A*. *lumbricoides* for evaluation of specific antibody responses, due to its association with helminth resistance and abundance in excretory/secretory fluids [6]. Recombinant ABA-1 was produced in the University of Cartagena as previously described [28]. This antigen was diluted in PBS and coated at 1 μg per well, respectively, on Nunc Maxisorb microtiter plates (Thermo Fisher Scientific, Waltham, MA, USA) at room temperature (RT) overnight and then blocked with blocking buffer (PBS pH 7.4, 2% BSA, 0.05% Tween 20). Plasma samples diluted 1:10 in blocking buffer were added and incubated for 2 hours at room temperature. Specific IgE was detected using a goat anti-human IgE antibody horseradish peroxidase conjugated (Bethyl Laboratories, Cat N° A80-108P, Texas, USA). For the detection of specific IgG, goat anti-human IgG-peroxidase (Jackson Immuno Research Europe Ltd, Cambridge shire, UK) was used. For specific IgG1 and IgG4 subclass detection, biotinylated anti-human IgG1 (clone: G17-1 RUO, BD bioscience, San Jose, CA, USA) or biotinylated anti-human IgG4 RJ4 Abs (Abingdon Health, York, UK) were used followed by incubation with streptavidin-peroxidase (Sigma-Aldrich, St. Louis, MO, USA). For specific IgG2 and IgG3 subclass detection, mouse anti-human IgG2 (clone: MH162-1, HP 6014, Sanquin, Amsterdam, the Netherlands) and mouse anti-human IgG3 (clone: MH163-1, HP 6095, Sanquin, Amsterdam, the Netherlands) were labeled with biotin (Sigma-Aldrich, St. Louis, MO, USA) and used as the primary detection antibodies. The colorimetric reaction was developed using tetramethylbenzidine (TMB) substrate (Thermo Fisher Scientific, Waltham, MA, USA) and the reaction was stopped with 1M H_2_SO_4_ sulfuric acid at OD450 nm. Plates were read at 450 nm by a Mithras LB 940 spectrophotometer (Berthold Technologies, Bad Wildbad, Germany). Serum from ten European donors without a history of *A*. *lumbricoides* infection were also tested for anti-ABA-1 IgG as a non-exposed population control.

### Statistical analysis

Most of the statistical analyses was performed with SPSS version 13.0 (Chicago, IL, USA). Since most of the variables did not have a Gaussian distribution, non-parametric methods were used for analyses. The bivariate tests included Pearson’s Chi-square to compare categorical variables and the Mann Whitney or Kruskal Wallis test to compare continuous variables between two or more groups, respectively.

The numerical data used in all figures are included in S1 Data.

## Supporting information

S1 Table Fluorescent coupled monoclonal antibodies used for Breg cell identification. (DOCX)

S1 Fig Different stimuli for testing IL-10 production in B cells. Results derived from 6-day cultures of PBMC isolated from patients with ascariasis.
(TIF)

S2 Fig Percentage of IL-10+ among total B cells or Breg cell subsets. Relative numbers of IL-10+ events among CD19- CD3- live cells (LB), CD25+CD71+CD73- LB (BR1), CD1hi LB, CD5+ LB, CD1hiCD5+ LB or CD24hiCD38hi LB are shown. Mean number is shown above each dot column. **p<0.01.
(TIF)

S3 Fig Specific IgG to ABA-1 in European residents and the sample population living in Santa Catalina, Colombia. Median IgG O.D. levels among three groups were compared with Kruskal Wallis test.
(TIF)

S4 Fig Cytokine levels in peripheral blood mononuclear cells stimulated with CD-MIX (anti-CD3, anti-CD2, anti-CD28). (TIF)

S1 Data Excel spreadsheet containing, in separate sheets, the underlying numerical data for Figs 1B, 1C, 2B, 2C, 3A, 3B, 3C, 3D, 4A, 4B, 4C, 4D, 4E, 4F and 4G. (XLSX)
