# Supplementary material for: Human Ascaris infection is associated with higher frequencies of IL-10 producing B cells
Source: PLoS Negl Trop Dis. 2024 Sep 23;18(9):e0012520. doi: 10.1371/journal.pntd.0012520 (PMC11537421; doi:10.1371/journal.pntd.0012520)
Supplement: S1 Table — (DOCX) [file pntd.0012520.s001.docx]

**Supplementary Table S**1 Fluorescent coupled monoclonal antibodies used for Breg cell identification.

| **Marker** | **Label** | **Clone** | **Company** | **Isotype*** |
| --- | --- | --- | --- | --- |
| **CD71** | FITC | CY1G4 | Biolegend | mIgG2a, k |
| **CD1d** | PE | 51.1 | eBioscienes | mIgG2b, k |
| **CD25** | PE-CF594 | M-A251 | BD Biosciences | mIgG1, k |
| **CD38** | Percp/Cy5.5 | HB-7 | Biolegend | mIgG1, k |
| **IL-10** | PE-Cy7 | JES3-9D7 | Biolegend | rIgG1, k |
| **CD73** | APC | AD2 | Biolegend | mIgG1, k |
| **CD5** | AF700 | OTI7A7 | Novusbio | mIgG1, k |
| **viability dye** | eF780 |  | Thermo Fisher Scientific |  |
| **CD24** | Brilliant Violet 421 | ML5 | Biolegend | mIgG2a, k |
| **CD19** | Brilliant Violet 510 | HIB19 | Biolegend | mIgG1, k |
| **CD14** | APC Cy7 | 61D3 | eBioscienes | mIgG1, k |
| **CD3** | APC Cy7 | UCHT1 | eBioscienes | mIgG1, k |
| **CD16** | APC Cy7 | 3G8 | eBioscienes | mIgG1, k |

m: murine, r: rat, k: kappa
