# Supplementary figures and images for: Human Ascaris infection is associated with higher frequencies of IL-10 producing B cells
Source: PLoS Negl Trop Dis. 2024 Sep 23;18(9):e0012520. doi: 10.1371/journal.pntd.0012520 (PMC11537421; doi:10.1371/journal.pntd.0012520)

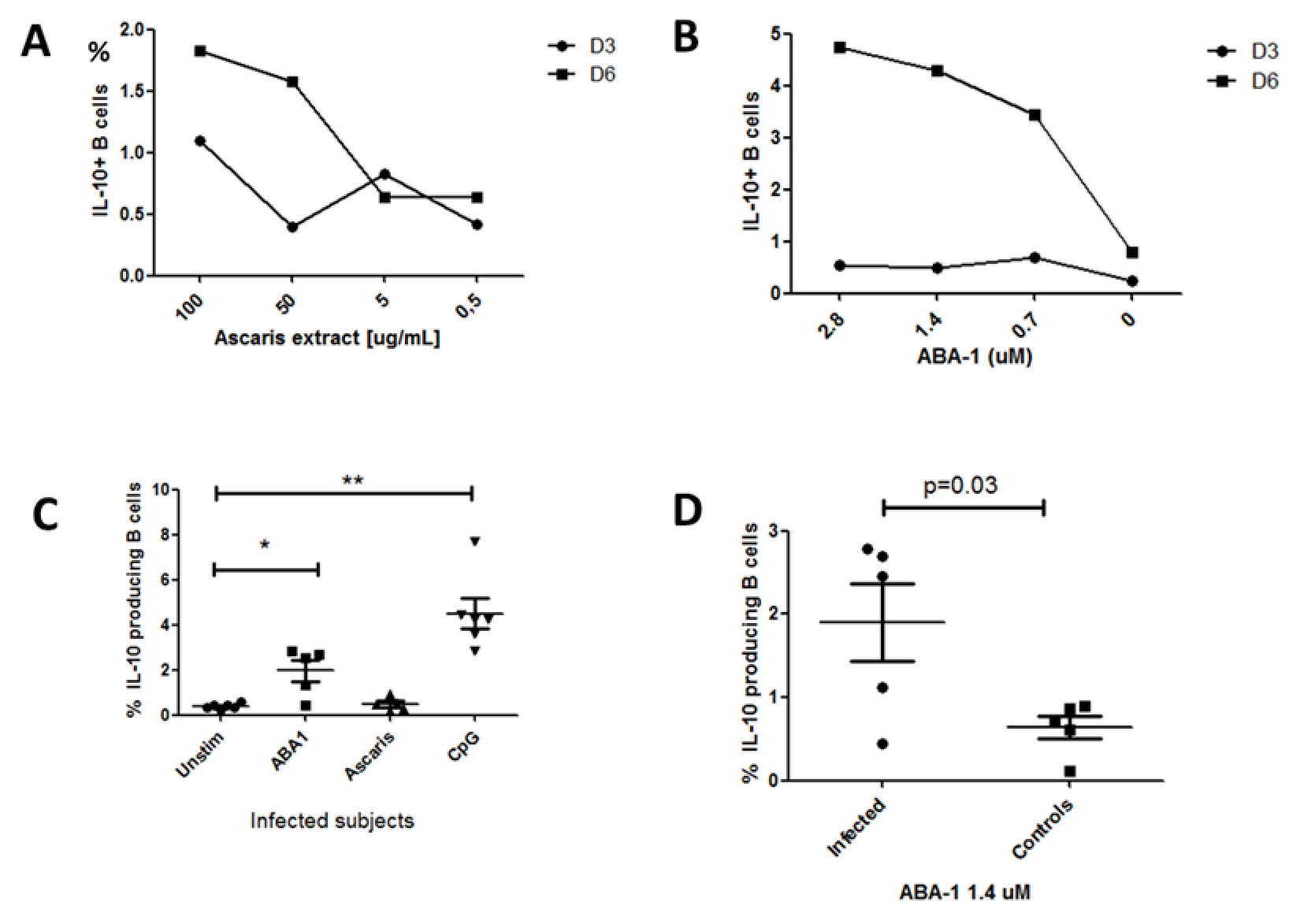

Supplement: S1 Fig — Results derived from 6-day cultures of PBMC isolated from patients with ascariasis. (TIF) [file pntd.0012520.s002.tif]

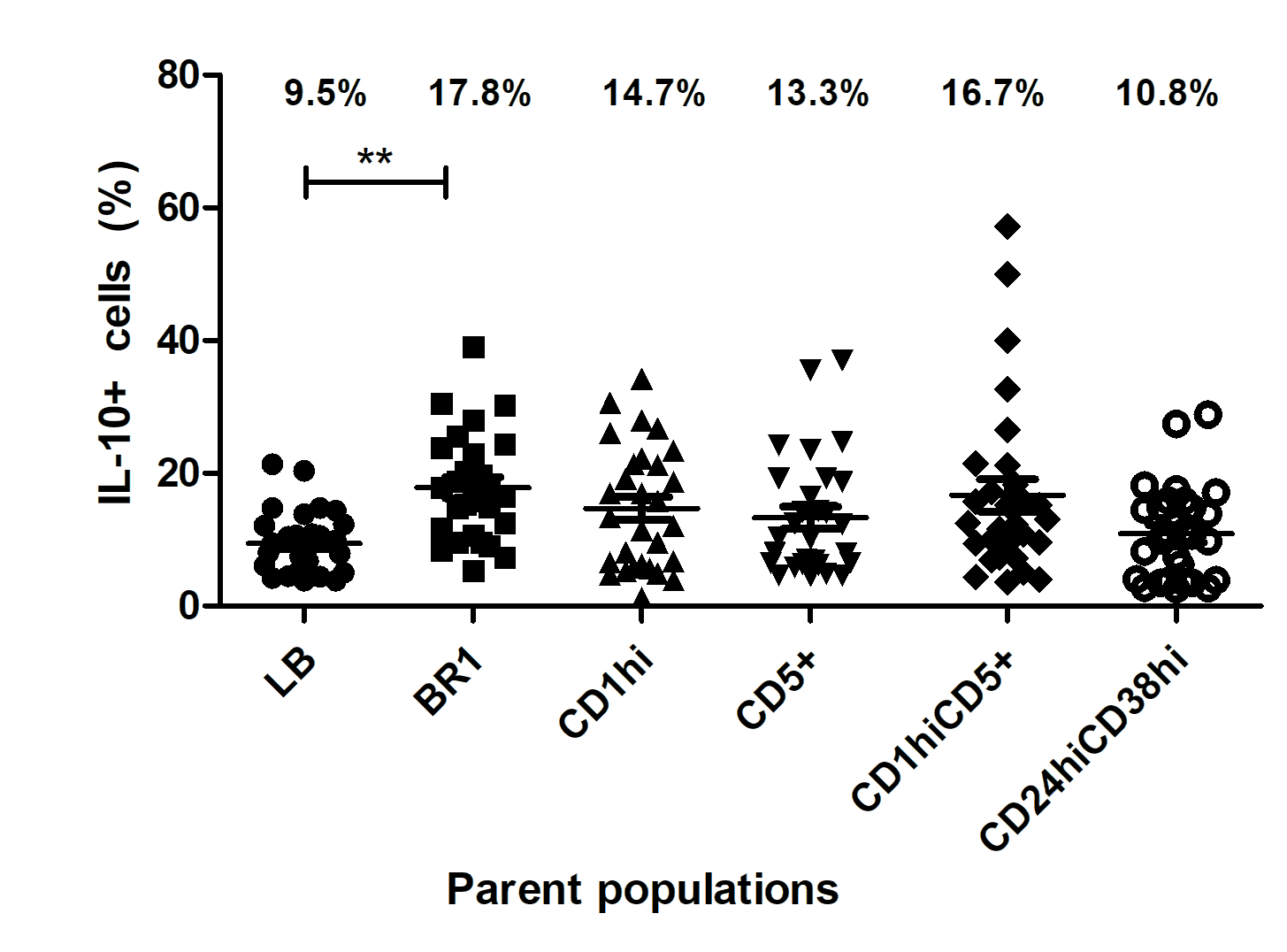

Supplement: S2 Fig — Relative numbers of IL-10+ events among CD19- CD3- live cells (LB), CD25+CD71+CD73- LB (BR1), CD1hi LB, CD5+ LB, CD1hiCD5+ LB or CD24hiCD38hi LB are shown. Mean number is shown above each dot column. **p<0.01. (TIF) [file pntd.0012520.s003.tif]

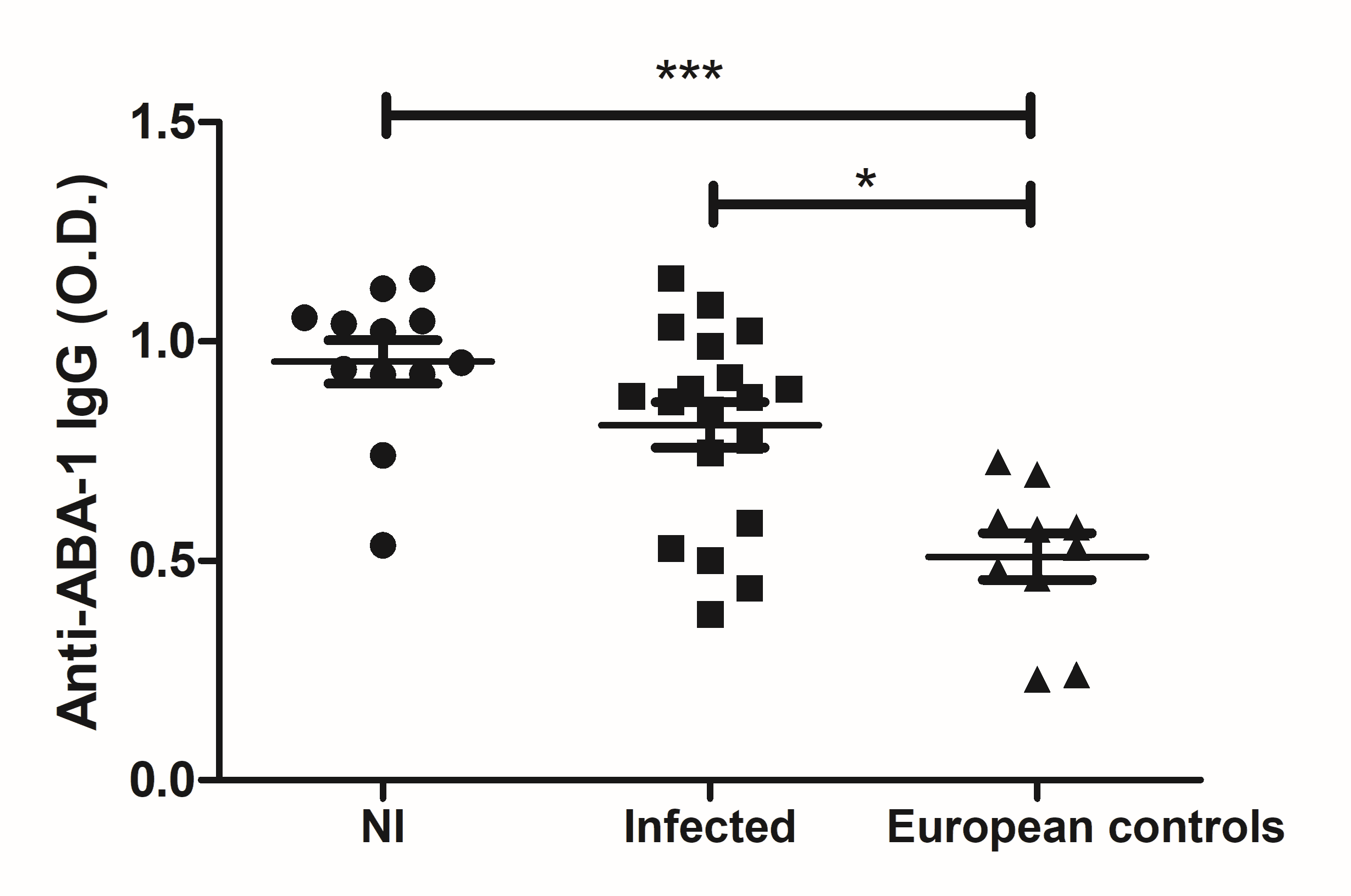

Supplement: S3 Fig — Median IgG O.D. levels among three groups were compared with Kruskal Wallis test. (TIF) [file pntd.0012520.s004.tif]

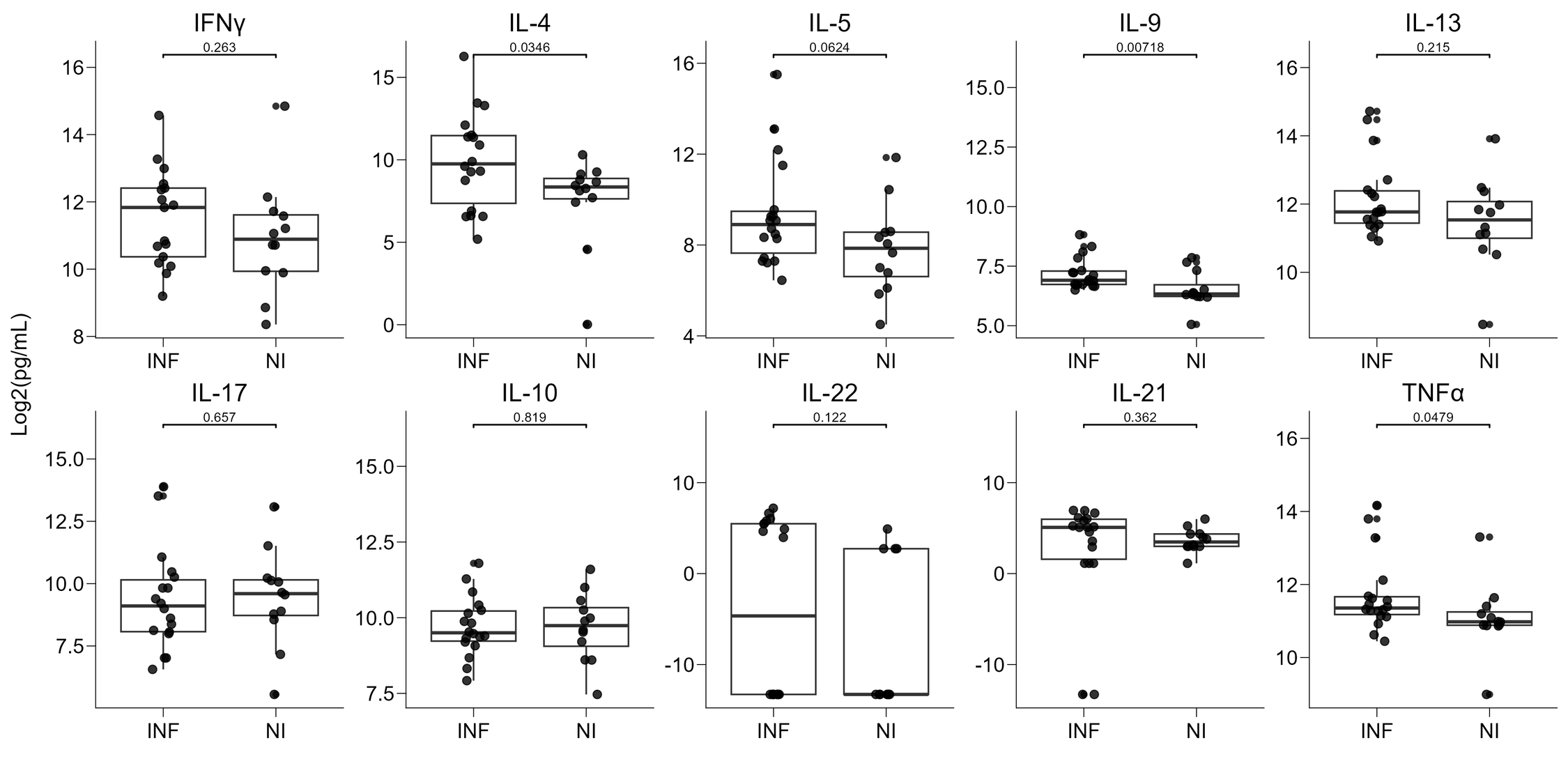

Supplement: S4 Fig — (TIF) [file pntd.0012520.s005.tif]
